# Multifocal Equine Influenza Outbreak with Vaccination Breakdown in Thoroughbred Racehorses

**DOI:** 10.3390/pathogens7020043

**Published:** 2018-04-17

**Authors:** Sarah Gildea, Marie Garvey, Pamela Lyons, Rachel Lyons, Jacinta Gahan, Cathal Walsh, Ann Cullinane

**Affiliations:** 1 Virology Unit, The Irish Equine Centre, Johnstown, Naas, Co. Kildare W91 RH93, Ireland; sarahgildea@hotmail.com (S.G.); MGarvey@irishequinecentre.ie (M.G.); PLyons@irishequinecentre.ie (P.L.); RLyons@irishequinecentre.ie (R.L.); JGahan@irishequinecentre.ie (J.G.); 2 Department of Mathematics and Statistics, University of Limerick, Castletroy, Limerick V94 T9PX, Ireland; Cathal.Walsh@ul.ie

**Keywords:** equine influenza Clade 2, TaqMan RT-PCR, vaccination breakdown, thoroughbred racehorses

## Abstract

Equine influenza (EI) outbreaks occurred on 19 premises in Ireland during 2014. Disease affected thoroughbred (TB) and non-TB horses/ponies on a variety of premises including four racing yards. Initial clinical signs presented on 16 premises within a two-month period. Extensive field investigations were undertaken, and the diagnostic effectiveness of a TaqMan RT-PCR assay was demonstrated in regularly-vaccinated and sub-clinically-affected horses. Epidemiological data and repeat clinical samples were collected from 305 horses, of which 40% were reported as clinically affected, 39% were identified as confirmed cases and 11% were sub-clinically affected. Multivariable analysis demonstrated a significant association between clinical signs and age, vaccination status and number of vaccine doses received. Vaccine breakdown was identified in 31% of horses with up to date vaccination records. This included 27 horses in four different racing yards. Genetic and antigenic analysis identified causal viruses as belonging to Clade 2 of the Florida sublineage (FCL2). At the time of this study, no commercially available EI vaccine in Ireland had been updated in line with World Organisation for Animal Health (OIE) recommendations to include a FCL2 virus. The findings of this study highlight the potential ease with which EI can spread among partially immune equine populations.

## 1. Introduction

Despite a comprehensive government-funded surveillance programme, mandatory vaccination of mobile equine populations and annual World Organisation for Animal Health (OIE) recommendations regarding vaccine composition, equine influenza (EI) remains one of the principal respiratory diseases affecting horses in Ireland, as it is in many countries around the world. Virus strains circulating in Europe during the past decade have primarily been identified as Clade 2 of the Florida sublineage of the American lineage (FCL2) [1,2,3,4,5,6,7,8]. Outbreaks involving Clade 1 (FCL1) viruses have also been identified, however, to a more limited extent [1,2,3,6,7]. The failure of companies to update vaccines with epidemiologically-relevant virus strains facilitates disease spread even among well-vaccinated populations [9,10,11,12,13]. This, in conjunction with other host and environmental risk factors, results in a potential threat to equine industries in countries where participation in equestrian events is of fundamental economic importance.

In Ireland, the majority of non-thoroughbred (non-TB) leisure horses and ponies are not vaccinated. This is in contrast to sport horses competing at Fédération Equestre Internationale (FEI) events and the thoroughbred (TB) racehorse population where mandatory vaccination policies have been in place since 1981. The focus of this study was to carry out a detailed epidemiological examination of EI activity in Ireland in 2014 when the virus affected more premises, both non-TB and TB, than any other outbreak since the turn of the century. This involved extensive investigations on affected premises with particular emphasis on the identification of host and epidemiological factors, which may have contributed to virus spread and subsequent vaccine breakdown among horses in TB racing yards. Furthermore, this study also sought to examine the diagnostic effectiveness of a TaqMan RT-PCR assay in a partially-immune population.

## 2. Results

### 2.1. Overview

**Figure 1:**
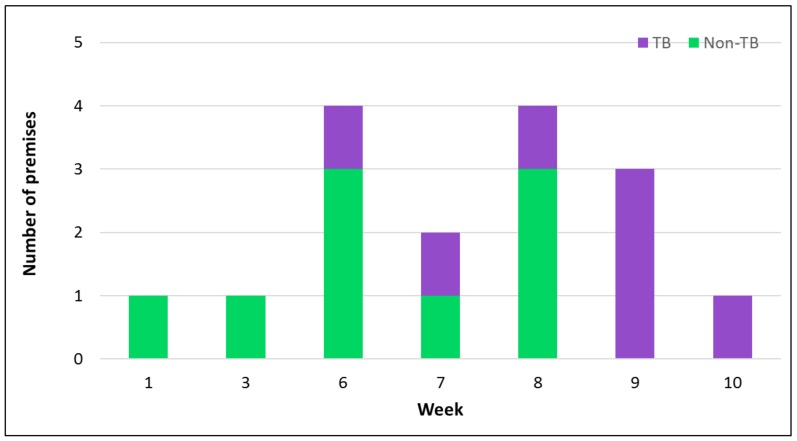
Number of premises and population affected from the week commencing 15 October 2014. TB, thoroughbred.

Equine influenza was diagnosed on three premises between 30 January and 17 February 2014 and on a further 16 premises between 22 October 2014 and 7 January 2015 (Table 1, Figure 1). Disease occurred among non-TB horses/ponies and TB horses on a variety of premises including four racing yards. Outbreaks affected premises in eight different counties with multiple outbreaks in some counties (Wexford n = 2, Tipperary n = 2, Kildare n = 2, Kilkenny n = 2, Clare n = 3, Meath n = 6). Farm investigations involving 305 horses on 18 of the 19 premises affected were undertaken. Serial collection of clinical samples was carried out on average every 7.2 ± 0.44 SE days on up to five occasions (average three samplings per premises). Of the 305 horses, 121 (40%) were reported clinically affected by the person responsible for the day to day management of the horses, 118 (39%) were identified as confirmed cases and 33 (11%) were sub-clinically affected.

### 2.2. Diagnosis

Veterinary intervention was sought on average 8.3 ± 1.45 SE days after the onset of clinical signs. Where veterinary intervention was ≤7.2 ± 1.17 SE days, a positive correlation between confirmed cases and the incidence of clinical signs was established (*p* = 0.001). Where no correlation existed, a significant delay in veterinary intervention was noted (≥12.5 ± 1.89 SE days).

Initial diagnosis on all premises was made by real-time RT-PCR using a primer probe-based assay on an ABI 7500 thermal cycler platform [14]. Retrospective testing of 1111 nasopharyngeal swabs collected during previous outbreaks in Ireland (2007–2012) demonstrated that this assay had equivalent specificity to a LightCycler SYBR Green assay [15] originally used for diagnostic testing [4,16]. However, an increase in assay sensitivity was demonstrated using the primer probe assay, when 32% of the 1111 nasopharyngeal swabs were identified as RT-PCR positive compared to 16% when using the LightCycler SYBR Green assay. Discordant samples generally had high cycle threshold (Ct) values, thus suggesting that the TaqMan assay was of superior sensitivity for the detection of low levels of viral nucleic acid (data not shown). These results were not considered to be false positives as the diagnostic specificity of the TaqMan assay was determined by the testing of 250 nasopharyngeal swabs from populations of horses free of equine influenza. No RT-PCR-positive results were obtained in these uninfected horses (data not shown).

Seven hundred and twenty six nasopharyngeal swabs were collected from 305 horses during this study. One hundred and thirty five of the 726 (19%) nasopharyngeal swabs collected tested positive by RT-PCR. Thirty four of the 135 (25%) were collected from 28 horses with an up to date vaccination record in accordance with the Turf Club rules and the vaccine manufacturers’ recommendations, i.e., a primary course of three vaccinations and subsequent boosters at not more than 12-month intervals. Of the RT-PCR-positive horses identified, those with no or incomplete vaccination records tested positive 1.4 ± 0.06 SE times and vaccinated horses tested positive 1.2 ± 0.09 SE times during consecutive weekly sampling.

Virus isolation was attempted on 29 nasopharyngeal swabs with a Ct value ≤28, and virus was isolated from 10 (34%). The average Ct value of nasopharyngeal swabs from which virus was isolated was 22.7 ± 0.99 SE (range 18–26). Eight of the 10 viruses were isolated on the first passage. Four of the 10 viruses were isolated from three horses with up to date vaccination histories. Virus was isolated on two occasions during a 72-h period from a vaccinated four-year-old gelding. This horse had received its fourth vaccine dose to five months prior to developing clinical signs and was the index case in the first TB premises affected during the outbreak (Premises 8). 

### 2.3. Factors Contributing to Disease Spread

All outbreaks occurred during the winter or autumn months and primarily following the return of horses from an equestrian event or the introduction of a new arrival. The suspected sources of infection were local hunt meets (seven premises), a visit to an equestrian centre/livery yard (two premises), a race meeting (one premises) and an international show (one premises). Outbreaks also occurred following the introduction of a new arrival on five premises. Three of these premises introduced young TB horses from public sales. There was no significant difference observed in the average period between exposure to the suspected source and onset of clinical signs on premises where the outbreak had occurred following recent movement (2.5 ± 0.37 SE days) or following the introduction of a new arrival (2.0 ± 0.55 SE days). Of the 16 premises from which the likely source of infection was identified, the travelling horse was the index case in 14. 

The average age of horses included in this study was 6.3 ± 5.4 SE years. Clinically-affected horses ranged from 6 months–16 years of age. Multivariable analysis demonstrated a significant difference between the age of horses that were clinically affected (4.2 ± 3.6 SE years) compared to those that remained healthy (7.5 ± 5.9 SE years) (*p* < 0.001). No association between gender and the incidence of clinical signs was established. 

The vaccination status of horses on each of the 19 premises is summarised in Table 1. A significant association between clinical signs and vaccination status (*p* < 0.001) was established. Two hundred and seven of the 305 (68%) horses in this study were unvaccinated or had an out of date vaccination history, of which 91 (44%) developed clinical signs. Of 98 horses that had up to date vaccination records, clinical signs were observed in 30 (31%). Vaccination breakdown was observed among horses last vaccinated with all four commercially available products (Table 2). No significant difference in vaccine product or time since last vaccination was identified. However, in vaccinated horses, there was a significant association between the absence of clinical signs and the total number of vaccine doses they had received prior to the outbreak (*p* = 0.035).

### 2.4. Serological Data

Serial samples were collected from 248 of the 305 (81%) horses included in this study. Sixty of the 248 horses, the majority of which were located on Premises 14 and 16, received booster vaccinations in the face of the outbreak and thus were excluded from the analysis of seroconversions. Of the remaining 188, 35 (19%) seroconverted to the FCL2 virus A/eq/Meath/1/07 by single radial haemolysis (SRH) and 27 (14%) seroconverted by haemagglutination inhibition (HI). Twenty four (13%) horses seroconverted in both tests. Twenty six (14%) horses that seroconverted by one or both methods tested RT-PCR positive. Of 43 horses that were RT-PCR positive and failed to seroconvert, 41 had a mean SRH antibody level of 191.3 ± 7.29 SE mm^2^ on initial sampling. Thus, these samples were probably collected after exposure to virus and post seroconversion.

#### Virus Characterisation

**Figure 2:**
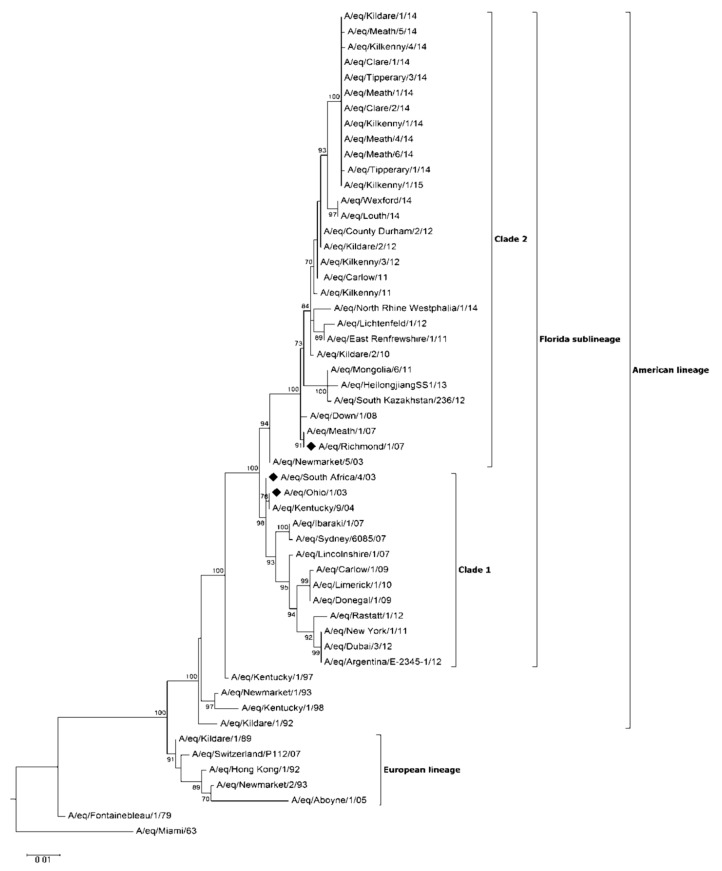
Phylogenetic tree of HA1 nucleotide sequences. Phylogenetic analysis of HA1 nucleotide sequences encoded by equine influenza viruses. The maximum likelihood tree was created by Mega Version 6. Bootstrap values were obtained after 1000 replicates and are shown at major nodes. Phylogenetic groups are shown by continuous bars on the right, as indicated. Representative viruses from each of the 13 premises on which HA sequence data were obtained are included. A/eq/Kilkenny/1/14 and A/eq/Kilkenny/4/14 were identified on a single premises. ♦ represents the strains included in the vaccines.

The complete HA gene sequence for 27 viruses identified on 13 of the 19 premises was determined in addition to the partial sequence obtained for a further two viruses (Figure 2, Figure S1). Analysis of the sequence data indicated that all viruses belonged to FCL2. All viruses had conserved amino acid substitutions P103L, V112I, A144V (antigenic site A), E291D, V300I in the HA1 gene when compared to A/eq/Meath/1/07 (Table 3). With the exception of V300I, all substitutions have been observed among EI viruses previously identified in Ireland [3,4]. V300I, not previously identified among Irish viruses, was noted among EI viruses, which circulated in the U.K. in 2013 [8]. Within the Irish viruses, a further two amino acid substitutions occurred in the HA1 gene (S6N, R208G), each on a single premises. Amino acid substitution S6N was shared among the final five of eight viruses identified on Premises 14 (including two viruses with partially-sequenced HA genes). This change was first observed in a nasopharyngeal swab collected from a subclinically-affected three-year-old TB gelding nine days after the onset of clinical signs in the index cases. This horse subsequently developed clinical signs within 24 h of sample collection. This amino acid substitution, not present in the initial three viruses identified on this premises or previously in any other Irish virus was observed in FCL1 viruses responsible for the first EI outbreak in Malaysia in nearly 40 years (A/eq/Malaysia/M201-1/2015, A/eq/Malaysia/ M201-2/2015) [17]. R208G was observed in a single EI virus identified in a racing yard in Co. Meath (Premises 16). This substitution, located at antigenic site D [18], was not previously been reported to the best of the authors’ knowledge.

Within the HA2 gene, all viruses in this study had the conserved amino acid substitution L187M compared to A/eq/Meath/1/07 (Table 3). A further three amino acid changes in the HA2 were observed within the Irish viruses. A113T occurred in A/eq/Wexford/14, and A/eq/Louth/14. C199Y was observed in two viruses identified in Tipperary (A/eq/Tipperary/1/14, A/eq/Tipperary/2/14), while G204S was observed in three viruses identified in Meath (A/eq/Meath/1/14, A/eq/Meath/2/14, A/eq/Meath/3/14) (Figure S1). With the exception of C199Y, all amino acid changes have previously been reported [8].

**Figure 3:**
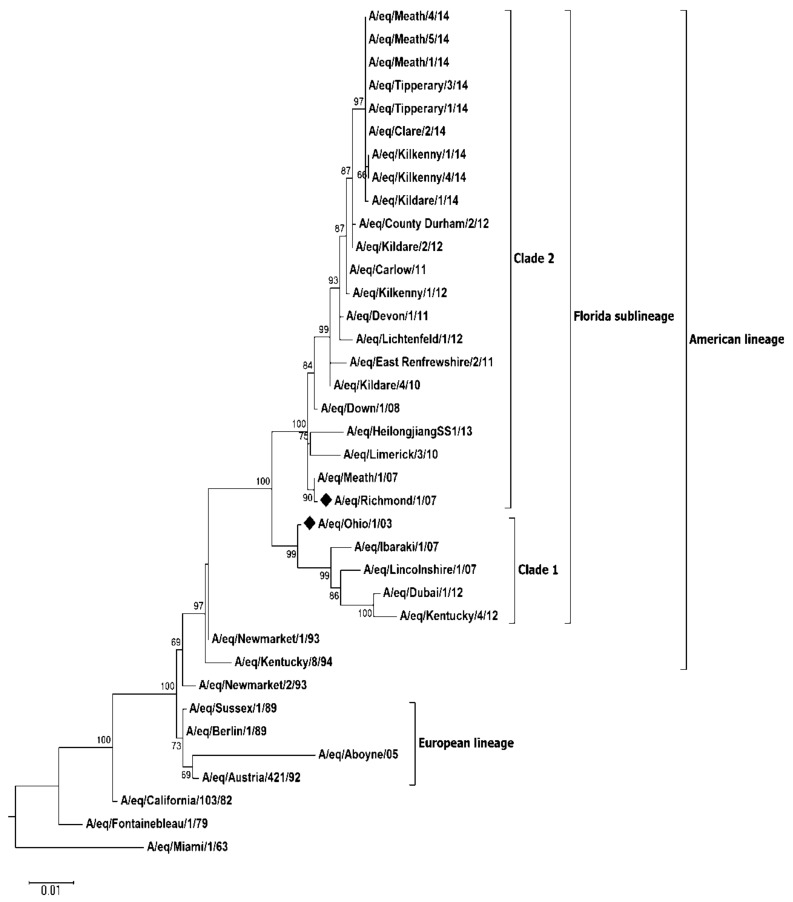
Phylogenetic tree of NA nucleotide sequences. Phylogenetic analysis of NA nucleotide sequences encoded by equine influenza viruses. The maximum likelihood tree was created by Mega Version 6. Bootstrap values were obtained after 1000 replicates and are shown at major nodes. Phylogenetic groups are shown by continuous bars on the right, as indicated. Representative viruses from each of the eight premises on which NA sequence data were obtained are included. A/eq/Kilkenny/1/14 and A/eq/Kilkenny/4/14 were identified on a single premises. ♦ represents strains included in the vaccines.

Due to the high level of vaccine breakdown observed during the outbreak, phylogenetic analysis of the second major surface glycoprotein neuraminidase (NA), for 9 viruses identified on 8 of the 19 premises along with historic Irish FCL2 (A/eq/Meath/1/07, A/eq/Down/1/08, A/eq/Kildare/4/10, A/eq/Carlow/11, A/eq/Kildare/2/12, A/eq/Kilkenny/1/12) and FCL1 viruses (A/eq/Limerick/3/10), identified during previous outbreaks, was also performed (Figure 3, Supplementary Figure S2). Similar to the HA gene, the topology of the NA phylogenetic tree grouped all recent viruses identified during this study within FCL2 and displayed clear separation between the pre-divergent, European, American and FCL1 sublineages. Conserved substitutions at residues 25, 42, 109, 410, 415 and 434 were observed in current circulating viruses when compared to A/eq/Meath/1/07 (Table 4). All of these changes have previously been reported among EI viruses circulating in the U.K. [5,8]. Additional substitutions within Irish viruses were also identified at residues 12 (A/eq/Kilkenny/1/14, A/eq/Kilkenny/4/14) and 22 (A/eq/Kildare/1/14). S12F was previously observed in the historic FCL2 virus A/eq/Newmarket/5/03 [5,9]. Amino acid substitution V22I has been reported among FCL2 viruses circulating in the U.K. [8] and Kazakhstan [19].

Antigenic characterisation with strain-specific ferret antisera confirmed the classification of A/eq/Clare/2/14 as belonging to FCL2 (Table 5). This virus exhibited more than a four-fold greater antibody titre when tested using FCL2 antisera compared to when using antisera raised against FCL1 viruses. The lowest antibody titres were observed using antisera raised against the European reference virus A/eq/Newmarket/2/93.

## 3. Discussion

In 2014, there was an increase in influenza activity in Europe, particularly in the U.K. and Ireland [20]. Equine influenza was identified among horses and ponies on a total of 12 non-TB and seven TB premises in Ireland. Initial diagnosis was made on three non-TB premises in January and February. This followed an absence of any confirmed EI activity in Ireland in 2013. During October and early November, a further four non-TB premises were affected. On 23 November, clinical signs were observed and EI was diagnosed in a TB four-year-old gelding, which had been vaccinated in accordance with the Turf Club Rules (Premises 8). This horse was a new arrival on the Curragh in Co. Kildare where the majority of TB training yards and Ireland’s premier racetrack are located. It had recently travelled from a non-TB premises in the west of Ireland. A further six TB premises were affected within the coming weeks including four racing yards. A similar pattern of virus spillover from non-TB to TB populations was observed in Ireland during a large outbreak in 1989, which originated at the Royal Dublin Society horse show and spread extensively throughout the country, affecting several racing yards [21]. 

The congregation of horses at any equestrian event is conducive to virus spread [4,16,22,23], and in this outbreak, EI occurred on 11 of 18 premises following attendance at such events. The risk associated with failing to isolate horses following recent movement or the introduction of a new arrival with an unknown immunological status [1,2,4,5,16] was evident in all the premises investigated in this study. Seven of the 11 premises were affected following the attendance of horses at a hunt meeting. Hunting has previously been implicated in EI virus spread [4] and may contribute to the seasonal pattern of EI observed in this and previous outbreaks [16]. The hunting “season” falls between November and April, and exposure of intrapulmonary airways to cold air [24] coupled with the strenuous physical activity may increase the horses’ susceptibility to EI [25], a situation exacerbated by the lack of a compulsory vaccination policy.

Of further concern is that EI occurred on three premises following the attendance of horses at a TB breeze up sale, an international horse show and a race meeting where up to date vaccination is required. During 2014 in Ireland, vaccine breakdown was identified in 30 horses vaccinated with all four commercially available products. This number was significantly higher than the three cases reported in the U.K. during the same period [8]. Morbidity ranged from 17–100% on the 18 affected premises investigated. Analysis of passports on non-TB premises indicated that of 149 horses, only 13 (9%) had an up to date vaccination records and 59 (40%) developed clinical signs. The incidence of clinical signs among TB horses was 62/156 (40%), i.e., identical to that observed among the non-TB population even though 98/156 (63%) of TB horses included in this study had an up to date vaccination record. Virological protection in vaccinated horses has been demonstrated to correlate with the degree of antigenic relatedness between the vaccine strain and the challenge virus [9,26,27]. FCL2 viruses have been circulating in Europe since 2003 [28], and although FCL1 viruses were identified in Ireland in 2009 and 2010, only FCL2 viruses have been identified since [3,4]. The current recommendation to include FCL2 and FCL1 representative viruses in EI vaccines was initially made by the OIE Expert Surveillance Panel in 2010 [29]. No updated vaccine was available at the time of last vaccination for horses included in this study. An updated ProteqFlu (Merial) recombinant vaccine expressing the HA genes of FCL1 (A/eq/Ohio/03) and FCL2 (A/eq/Richmond/07) viruses became available in Ireland towards the end of 2014 [30].

Analysis of HA sequence data indicated that all viruses identified during the Irish outbreaks belonged to FCL2. In Europe in recent years, two subpopulations of FCL2 viruses have been identified with amino acid substitutions at position 144 or 179 in the HAI gene [8]. All viruses characterised in the current study contained A144V similar to viruses identified during previous Irish outbreaks [4] and genetically distinct from an I179V group of EI viruses, which have circulated in Germany, Italy and in some parts of the U.K. [8]. The biological significance of the substitutions merits investigation. No antigenic difference between these two FCL2 subgroups has been demonstrated using the HI assay and post-infection ferret antisera [8]. However, this is in contrast to results observed using an egg-based virus neutralization test and equine antisera raised against the Argentinian lineage vaccine strain A/eq/La Plata/93 [31]. 

Both HA and NA are important surface glycoproteins of influenza virus, but the selection of vaccine strains has traditionally focused only on the HA. Due to the significant increase in vaccine breakdown observed in the current study, sequence analysis of the NA gene was carried out. Similar to the results obtained for the HA gene, the NA phylogenetic tree grouped all recent Irish viruses’ within FCL2. Of note however, the NA of A/eq/Limerick/3/10 a FLC1 [3] virus was grouped within FCL2 NA subgroup, indicating that reassortment had taken place between the clades. Such reassortment had been reported previously in the U.K. [1,5].

Similar to previous findings, a correlation between age, time since last vaccination and number of vaccine doses received was also established [4,16]. Maternal antibodies in foals born to seropositive mares generally persist for up to six months; however, in some cases, these may be more durable [32,33,34]. In a comparative antibody study, which examined the antibody levels in different equine populations in Ireland, TB weanlings (age 6–10 months) were identified as the most susceptible [35]. In this study, virus infection among TB weanlings in Ireland was identified for the first time (Premises 9, 10, 14). Vaccination of weanlings is currently not a requirement for attendance at TB sales irrespective of immunological status. Weanlings on two of the three premises had recently arrived from large TB sales in Ireland (Premises 9) and the U.K. (Premises 10). The index case on both premises was unvaccinated and seronegative on initial sampling. 

Among vaccinated horses, infection occurred on average 239 ± 19.2 SE days (range 42–364 days), i.e., approximately eight months following last vaccination. This was observed in horses with an average age of 3.6 ± 0.37 SE years (range 1–8 years). The poor durability of the response of young horses to vaccination especially early in their vaccination career is well documented [36,37,38]. Mathematical models based on data derived from experimental challenge studies, as well as field data indicate that six-month, rather than annual, booster vaccination reduces the risk of EI infection in young racehorses [39]. Of 25 index cases identified in this study, seven had an up to date vaccination history. 

Laboratory testing is necessary to confirm a clinical diagnosis of EI. The real-time RT-PCR primer probe-based assay was the primary method of diagnosis during this study [14]. This assay was extensively used to detect EI in naïve horses in Australia in 2007 [40]. The current study demonstrated its diagnostic effectiveness in seropositive partially-immune and regularly-vaccinated horses. Twenty five percent of RT-PCR positive nasopharyngeal swabs identified during this study were collected from horses with up to date vaccination records. Of 135 RT-PCR positive nasopharyngeal swabs, 125 (93%) had accompanying serum samples. On serological analysis, 22 (18%) horses were seronegative, and the 103 (82%) seropositive horses had a mean FCL2 SRH antibody level of 181 ± 4.4 SE mm^2^. The maximum FCL2 antibody level detected in an RT-PCR positive horse was 297 mm^2^. These high SRH values were not necessarily representative of the horses’ serological status pre-infection, but they indicate that RT-PCR is a sensitive diagnostic technique in seropositive horses.

Read et al. (2011) reported that when monitoring immunologically-naïve horses in Australia, virus was detected by RT-PCR from a single horse for a maximum period of 34 days [41]. The maximum period for which virus was detected in two unvaccinated horses during the current study was 12 days (Premises 1 and 3). Both of these horses, a four-year-old gelding and a seven-year-old mare, had FCL2 SRH antibody levels >150 mm^2^ on initial sampling. On a single premises, virus was detected for a maximum period of 21 days (Premises 3). On four premises, virus was detected up to one week after the last seroconversion was identified (Premises 12, 14, 15, 16). 

The serological examination of paired acute and convalescent sera for the diagnosis of EI is used by many laboratories as an adjunct test to RT-PCR/virus isolation. Hemagglutination inhibition assays are routinely used with detergent-treated viral antigens to improve assay sensitivity. However, SRH tests with untreated antigens have been shown to be more reproducible between laboratories, and a strong correlation between SRH antibody level and protection has been established [42,43,44,45]. Thus, SRH is the serological test of choice for measuring vaccine efficacy. Few studies, however, have compared EI serological techniques during outbreaks [46,47]. Similar to the findings by Morley et al. [46], a good correlation between the HI and SRH test was observed in this study, although SRH demonstrated greater diagnostic sensitivity. The use of improved diagnostics greatly contributes to the detection of subclinical infection with 33 (11%) horses included in this study identified as subclinically affected. Twenty six subclinically-infected horses were identified by RT-PCR only, six by SRH only and one by RT-PCR, SRH and HI. Increased detection of subclinically-infected horses, which are shedding virus, is key to minimizing the risk of introducing EI to naïve populations through the international movement of horses.

Farm investigations carried out in the current study are a critical component to EI surveillance programmes. These investigations coupled with detailed examination of vaccination histories are an important component of the Department of Agriculture, Food and the Marine (DAFM)-funded EI surveillance programme in Ireland. They offer the opportunity to examine the pattern of disease spread among different equine populations in real time and provide data to the OIE Expert Surveillance Panel regarding the viruses circulating in the field and the effectiveness of current vaccines. This study also facilitated the evaluation of an RT-PCR diagnostic test for the detection of infection in vaccinated horses. This is pertinent as recently, the OIE together with the FEI and the International Federation of Horseracing Authorities (IFHA) has identified EI as one of six OIE listed diseases at risk of dissemination as a result of international competition horse movements. The availability of EI virus detection tests that are sufficiently sensitive to detect low quantities of virus shed by vaccinated competition horses is considered paramount for screening horses for freedom from infection and safeguarding not only the horses competing at an international event, but also the population of the host country.

## 4. Materials and Methods

### 4.1. Sample Collection and Clinical Histories

Nasopharyngeal swabs and clotted blood samples were collected from horses with clinical signs of acute respiratory disease and submitted to the Irish Equine Centre for diagnostic testing. The nasopharyngeal swabs were custom made for the Irish Equine Centre and after collection were placed in 5-mL containers of viral transport medium consisting of 4.7 mL phosphate-buffered saline (PBS) at a dilution of 1:100, 0.1 mL penicillin (5000 u/mL), 0.1 mL amphotericin B (250 mg/ mL) and 0.1 mL bovine foetal calf serum. Following a confirmed diagnosis of EI and in consultation with the attending veterinary surgeon/owner, a farm investigation was undertaken. Repeat sampling (nasopharyngeal swabs and clotted blood samples) of all horses on affected premises was undertaken on a weekly basis where practical, until all horses tested negative by RT-PCR. Clinical and vaccination histories were obtained from the attending veterinary surgeon and the personnel involved in the day-to-day management of the horses. On the premises where EI was diagnosed, morbidity was defined as the presence of at least one of the three most common clinical signs associated with the virus, i.e., nasal discharge, coughing and pyrexia [16]. A confirmed case was identified as any horse that tested positive by at least one of the following methods: RT-PCR, virus isolation and seroconversion [4]. 

### 4.2. RNA Extraction and Real-Time RT-PCR

RNA was extracted from 100-µL nasal secretions and 2 µL (20,000 copies) of Xeno RNA exogenous control with magnetic beads (MagVet^TM^ Universal Purification Kit) using the Kingfisher Flex 96 magnetic particle processor (Thermo Scientific, Waltham, MA, USA) in accordance with the manufacturer’s instructions. One-step real-time RT-PCR using a primer probe Spackman-based assay [14,40,48], which targets the matrix gene of influenza A virus, was carried out on an ABI 7500 Fast thermal cycler platform (Applied Biosystems, Foster City, CA, USA) using an AgPath-ID One-Step RT-PCR kit (Applied Biosystems, Foster City, CA, USA). Briefly, five microliters of purified nucleic acid were added to a 20-μL reaction mix containing 2X RT-PCR buffer, 0.08 ng tRNA (Sigma Aldrich, St. Louis, MO, USA), 0.32 µM of each primer, 0.08 μM of probe, 1 μL Xeno primer/probe assay and 25X RT enzyme and nuclease free H_2_O. One-step RT-PCR was carried out at 45 °C for 10 min, 95 °C for 10 min, followed by 40 cycles of 95 °C for 15 s and 60 °C for 45 s. Nasopharyngeal swabs with a *C*t value ≤40 were classified as positive.

### 4.3. Equine Influenza Antibody Detection and Quantification

Antibodies against A/eq/Meath/1/07 (FCL2) were detected by HI in accordance with the standard procedure [49]. A seroconversion between acute and convalescent samples was defined as a ≥4-fold increase in antibody titre. Antibodies against A/eq/Meath/1/07 were also measured by SRH as previously described [35]. Results were expressed in mm^2^, and a seroconversion was defined as a ≥25 mm^2^ increase in antibody level between paired samples [43]. A horse was designated seronegative if its serum exhibited no haemolytic activity.

### 4.4. Virus Isolation

Virus isolation was carried out on all nasopharyngeal swabs that had a Ct value of ≤28. Briefly, 0.1 mL nasopharyngeal secretions at neat, 10^−1^ and 10^−2^ dilutions were inoculated into the allantoic cavity of two 9–12-day-old eggs and incubated at 34 °C for 72 h. Eggs were subsequently chilled overnight (2–8 °C) and the allantoic fluid harvested and tested for haemagglutination (HA) activity using 1% chicken red blood cells [49]. Negative samples were passaged up to four times. 

### 4.5. Haemagglutinin and Neuraminidase Gene Sequencing

During the outbreak HA and NA gene sequencing was carried out on all nasopharyngeal swabs, which had a Ct value of ≤30. For historical isolates (A/eq/Meath/1/07, A/eq/Down/1/08, A/eq/Limerick/3/10, A/eq/Kildare/4/10 and A/eq/Carlow/1/11) where there was insufficient original sample available, the NA sequencing was performed using low passage (≤3) egg-grown isolates. Sequencing was performed using a SuperScript III One-Step RT-PCR System with Platinum Taq High Fidelity (Life Technologies, Carlsbad, CA, USA) in accordance with the manufacturer’s instructions. Gene specific primers were as follows: HA 5′AGCAAAAGCAGGGGATATTTC, HA 3′AGTAGAAACAAGGGTGTTTTTAACTATC, NA 5′AGTAGAAACAAGGAGTTTTT, NA 3′AGCAAAAGCAGGAGTTTAAA. Where the full HA gene sequence was not obtainable using a single primer pair, multiple HA primers were used as previously described [3]. Where the full NA gene sequence was not obtainable using a single primer pair, sequencing was performed using M13 tagged primers [50]. One-step RT-PCR was carried out at 55 °C for 30 min, 94 °C for two minutes followed by 40 cycles of 94 °C for 60 s, 45–50 °C for 10 s, 60 °C for one minute with a final extension of 60 °C for five minutes. RT-PCR products were analysed on a 1% agarose gel stained with SYBR safe DNA gel stain (Invitrogen, Carlsbad, CA, USA) and purified using the QIAquick gel extraction kit (Qiagen, Hilden, Germany). Sequencing was performed by Qiagen Sequencing Services (Hilden, Germany) and GATC Biotech (Konstanz, Germany). Genetic analysis was undertaken using Seqman Version 5.01 software, DNASTAR, Madison, WI, USA. 

### 4.6. Genetic Sequence Analysis

Maximum likelihood (ML) phylogenetic trees for the nucleotide sequence encoding the HA1 (1009 nt) and the NA (1410 nt) genes were created using MEGA 6.0 software. Genetic distances were calculated using the Kimura two-parameter model, and statistical support for the tree topology was assessed by bootstrap resampling (1000 replicates) of the multiple alignments. Nucleotide and amino acid sequences alignments were constructed using BioEdit software package Version 7.2.5 (Ibis Therapeutics Inc., Carlsbad, CA, USA). All gene sequences included in the phylogenetic analysis (accession numbers included in Table S1) were obtainable from the NCBI database. 

### 4.7. Antigenic Characterisation

Haemagglutination inhibition testing using Clade-specific ferret antisera generated as previously described was carried out in accordance with the standard procedure [5]. Briefly, 4 HA units of each virus were tested with serial dilutions of ferret antisera raised against representative FCL1 (A/eq/South Africa/4/03 and A/eq/Donegal/1/09) and FCL2 (A/eq/Meath/1/07 and A/eq/Kildare/2/12) viruses. Each assay was carried out in duplicate and geometric mean titres calculated.

### 4.8. Statistical Analysis

A multilevel logistic regression model was carried out using open source package R Version 3.1.1 to identify factors that contributed to virus spread. Prevalence factors were analysed as independent variables, and those with statistical significance (*p* ≤ 0.05) were included in the model.

## Figures and Tables

**Table 1:** Equine influenza activity in Ireland 2014.

PID	Date	Location (County)	Method of Case Confirmation	Premises Type	Veterinary Intervention	Clinical Cases (%)	Confirmed Cases (%)	Up to Date Vaccination Records Available (%)
1	January 2014	Wexford	P 6, PS 2	Non-TB	14 days	14/14 (100%)	8/14 (57%)	0/14 (0%)
2	February 2014	Wexford	P 3, PS 1	Mixed non-TB	14 days	4/5 (80%)	4/5 (80%)	1/5 (20%)
3	February 2014	Louth	P 9, S 3, PS 2	Equestrian centre/livery yard	3 days	5/29 (17%)	14/29 (48%)	1/29 (3%)
4	October 2014	Meath	P 3	Non-TB	7 days	3/3 (100%)	3/3 (100%)	0/3 (0%)
5	November 2014	Tipperary	S 1, PS 4	Mixed non-TB	5 days	5/7 (71%)	5/7 (71%)	0/7 (0%)
6	November 2014	Tipperary	P 2, S 2, PS 5, PSV 1	Mixed non-TB	2 days	5/16 (31%)	10/16 (63%)	6/16 (38%)
7	November 2014	Clare	S 1, PS 1	Show jumping	4 days	3/13 (23%)	2/13 (15%)	4/13 (31%)
8	November 2014	Kildare	P 1, PSV 1	TB (mixed ages and disciplines)	1 day	2/5 (40%)	2/5 (40%)	5/5 (100%)
9	November 2014	Clare	PS 2	TB (mixed ages and disciplines)	17 days	12/30 (40%)	2/30 (7%)	0/30 (0%) (18/30 reported vaccinated) *
10	December 2014	Kildare	PS 1	TB public stud	3 days	3/3 (100%)	1/3 (33%)	0/3 (0%)
11	December 2014	Clare	PS 2, PSV 1	Non-TB	8 days	3/4 (75%)	3/4 (75%)	0/4 (0%)
12	December 2014	Meath	P 1	Non-TB	5 days	1/1 (100%)	1/1 (100%)	0/1 (0%)
13	December 2014	Dublin	P 6, S 2, PS 3	Equestrian centre/livery yard	16 days	8/32 (25%)	11/32 (34%)	1/32 (3%)
14	December 2014	Kilkenny	P 4, S 1, PS 2, PV 2, PSV 2	Racing yard/TB breeding farm	2 days	13/25 (52%)	11/25 (44%)	10/25 (40%)
15	December 2014	Meath	P 7	Riding school/livery yard	10 days	6/19 (32%)	7/19 (37%)	0/19 (0%)
16	December 2014	Meath	P 16, PS 1, PV 1	Racing yard	2 days	17/47 (36%)	18/47 (38%)	41/47 (87%)
17	December 2014	Meath	P 5, PV 1	Racing yard	6 days	3/13 (23%)	6/13 (46%)	10/13 (77%)
18	December 2014	Meath	P 4	Mixed non-TB	17 days	2/6 (33%)	4/6 (67%)	0/6 (0%)
19	January 2015	Kilkenny	P 3, S 2, PS 1	Racing yard	21 days	12/33 (36%)	6/33 (18%)	19/33 (58%)

Data presented include premises’ number = PID, date of diagnosis, location, method of case confirmation (P = RT-PCR positive, S = seroconverted, PS = RT-PCR positive and seroconverted, PV = RT-PCR positive and virus isolated, PSV = RT-PCR positive, seroconverted and virus isolated), type of premises, period before veterinary intervention following the onset of clinical signs in the index case, number of clinical cases, number of confirmed cases and vaccination status. Mixed = horses and ponies; * = no passports available.

**Table 2:** Equine influenza vaccine breakdown observed in horses with up to date vaccination records.

Last Vaccine Dose (Manufacturer)	No. of Horses (%)	Vaccine Breakdown (%)	No. of Days Since V2	No. of Days Since V3 or Subsequent Booster	Representative H3N8 American Lineage Vaccine Strain
Duvaxyn IET Plus (Elanco Animal Health)	43 (44%)	13/43 (30%)	124 (n = 1)	276 ± 27.7 SE (n = 12)	A/eq/Newmarket/1/93
Equip FT (Zoetis)	10 (10%)	5/10 (50%)	189 (n = 1)	267 ± 51.2 SE (n = 4)	A/eq/Kentucky/98
Equilis Prequenza Te (MSD Animal Health)	32 (33%)	9/32 (28%)	135 ± 33.5 SE (n = 2)	217 ± 35.7 SE (n = 7)	A/eq/South Africa/4/03 ^1^
ProteqFlu Te (Merial)	13 (13%)	3/13 (23%)	42 (n = 1)	257 ± 95.5 SE (n = 2)	A/eq/Ohio/03 ^2^
Total/average	98	30/98 (31%)	125 ± 25.9 SE (n = 5)	261 ± 19.6 SE (n = 25)	

No. = number; No. of days since V2 = the number of days since the horse received the second dose of the primary vaccination. No. of days since V3 = the number of days since the horse received the third dose of the primary vaccination. ^1^ Equilis Prequenza updated vaccine became available in Ireland in 2013 and includes Clade 1 Florida sublineage of the American lineage (FCL1) representative vaccine strain A/eq/South Africa/4/03 (previously contained A/eq/Newmarket/1/93). ^2^ ProteqFlu updated vaccine became available in Ireland in December 2014 and includes recombinant canarypox viruses expressing the haemagglutinin gene of A/eq/Ohio/03 (FCL1) and A/eq/Richmond/1/07 (FCL 2).

**Table 3:** Amino acid substitutions within the HA1 and HA2 genes between viruses identified and the Irish FCL2 representative virus A/eq/Meath/1/07.

	HA1							HA2			
*Animo Acid Number*	6	103	112	144	208	291	300	113	187	199	204
A/eq/1/Meath/07	S	P	V	A	R	E	V	A	L	C	G
A/eq/Wexford/14	.	L	I	V	.	D	I	T	M	.	.
A/eq/Louth/14	.	L	I	V	.	D	I	T	M	.	.
A/eq/Meath/1/14	.	L	I	V	.	D	I	.	M	.	S
A/eq/ Tipperary/1/14	.	L	I	V	.	D	I	.	M	Y	.
A/eq/Tipperary/3/14	.	L	I	V	.	D	I	.	M	.	.
A/eq/ Clare/1/14	.	L	I	V	.	D	I	.	M	.	.
A/eq/Kildare/1/14	.	L	I	V	.	D	I	.	M	.	.
A/eq/Clare/2/14	.	L	I	V	.	D	I	.	M	.	.
A/eq/ Kilkenny/1/14 *	.	L	I	V	.	D	I	.	M	.	.
A/eq/Kilkenny/4/14 *	N	L	I	V	.	D	I	.	M	.	.
A/eq/Meath/4/14	.	L	I	V	.	D	I	.	M	.	.
A/eq/Meath/5/14	.	L	I	V	G	D	I	.	M	.	.
A/eq/Meath/6/14	.	L	I	V	.	D	I	.	M	.	.
A/eq/Kilkenny/1/15	.	L	I	V	.	D	I	.	M	.	.

The table includes representative viruses from each of the 13 premises on which HA sequence data were obtained. Amino acid residues are numbered from the serine residue immediately downstream of the predicted signal sequence. Amino acid identity to the Irish FCL2 representative virus A/eq/Meath/1/07 is shown with a dot. * A/eq/Kilkenny/1/14 and A/eq/Kilkenny/4/14 were identified on the same premises.

**Table 4:** Amino acid substitutions within the NA gene between viruses identified and the Irish FCL2 representative virus A/eq/Meath/1/07.

	NA							
Animo Acid Number	12	22	25	42	109	410	415	434
A/eq/Meath/1/07	S	V	H	G	R	I	K	T
A/eq/Meath/1/14	.	.	N	C	K	V	R	S
A/eq/Tipperary/1/14	.	.	N	C	K	V	R	S
A/eq/Tipperary/3/14	.	.	N	C	K	V	R	S
A/eq/Kildare/1/14	.	I	N	C	K	V	R	S
A/eq/Clare/2/14	.	.	N	C	K	V	R	S
A/eq/Kilkenny/1/14 *	F	.	N	C	K	V	R	S
A/eq/Kilkenny/4/14 *	F	.	N	C	K	V	R	S
A/eq/Meath/4/14	.	.	N	C	K	V	R	S
A/eq/Meath/5/14	.	.	N	C	K	V	R	S

Includes representative viruses from each of the eight premises on which NA sequence data were obtained. Amino acid residues are numbered from the N-terminal methionine located at the beginning of the predicted signal sequence. Amino acid identity to the Irish FCL2 representative virus A/eq/Meath/1/07 is shown with a dot. * A/eq/Kilkenny/1/14 and A/eq/Kilkenny/4/14 were identified on the same premises.

**Table 5:** Antigenic characterisation of the 2014 isolate by haemagglutination inhibition assay using ferret antisera.

	Reference Ferret Antisera					
Reference Virus Strain	A/eq/NM/1/93 Am	A/eq/NM/2/93 Eu	A/eq/SA/4/03 FCL1	A/eq/DL/1/09 FCL1	A/eq/MH/1/07 FCL2	A/eq/KE/2/12 FCL2
A/eq/NM/1/93	**181**	32	181	256	724	1024
A/eq/NM/2/93	64	**256**	64	64	256	256
A/eq/SA/4/03	64	32	**1448**	1024	512	512
A/eq/DL/1/09	256	128	1024	**2896**	256	256
A/eq/MH/1/07	362	128	181	256	**724**	512
A/eq/KE/2/12	181	91	128	256	1024	**1024**
A/eq/Clare/2/14	181	91	128	256	1024	**1024**

A/eq/NM/1/93 = A/eq/Newmarket/1/93; Am = American lineage; A/eq/NM/2/93 = A/eq/Newmarket/2/93; Eu = European lineage; A/eq/SA/4/03 = A/eq/SouthAfrica/4/03; FCL1 = Florida sublineage Clade 1; A/eq/DL/1/09 = A/eq/Donegal/1/09; A/eq/MH/1/07 = A/eq/Meath/1/07; FCL2 = Florida sublineage Clade 2; A/eq/ KE/2/12 = A/eq/Kildare/2/12. Homologous titres are indicated in bold.

## Supplementary Materials

The following are available online at http://www.mdpi.com/2076-0817/7/2/43/s1: Table S1: Equine influenza viruses included in phylogenetic analysis. Figure S1: HA amino acid alignment. Figure S2: NA amino acid alignment.
