# Supplementary material for: Multifocal Equine Influenza Outbreak with Vaccination Breakdown in Thoroughbred Racehorses
Source: Pathogens. 2018 Apr 17;7(2):43. doi: 10.3390/pathogens7020043 (PMC6027538; doi:10.3390/pathogens7020043)
Supplement: Supplementary file 1 [file pathogens-07-00043-s001.zip › SupplementaryData/Supplementary Figure S1.docx]

**Supplementary Figure S1: HA amino acid alignment.** Alignment of the predicted HA amino acid sequences of Irish FCL2 strains identified in 2014/2015 against reference strain A/eq/Meath/1/07. Amino acid identity is represented with a dot.

**HA1**

10 20 30 40 50 60 70 80 90 100

....|....|....|....|....|....|....|....|....|....|....|....|....|....|....|....|....|....|....|....|

**A/eq/Meath/1/07**  **SQNPISNNNTATLCLGHHAVANGTLVKTISDDQIEVTNATELVQSISMGKICNNSYRILDGRNCTLIDAMLGDPHCDVFQYENWDLFIERSSAFSNCYPY** 100

**A/eq/Wexford/14**  **....................................................................................................** 100

**A/eq/Louth/14**  **....................................................................................................** 100

**A/eq/Meath/1/14**  **....................................................................................................** 100

**A/eq/Meath/2/14**  **....................................................................................................** 100

**A/eq/Meath/3/14**  **....................................................................................................** 100

**A/eq/Tipperary/1/14** **....................................................................................................** 100

**A/eq/Tipperary/2/14** **....................................................................................................** 100

**A/eq/Tipperary/3/14** **....................................................................................................** 100

**A/eq/Tipperary/4/14** **....................................................................................................** 100

**A/eq/Tipperary/5/14** **....................................................................................................** 100

**A/eq/Tipperary/6/14** **....................................................................................................** 100

**A/eq/Clare/1/14**  **....................................................................................................** 100

**A/eq/Clare/2/14**  **....................................................................................................** 100

**A/eq/Clare/3/14**  **....................................................................................................** 100

**A/eq/Clare/4/14**  **....................................................................................................** 100

**A/eq/Kildare/1/14**  **....................................................................................................** 100

**A/eq/Meath/4/14**  **....................................................................................................** 100

**A/eq/Meath/5/14**  **....................................................................................................** 100

**A/eq/Meath/6/14**  **....................................................................................................** 100

**A/eq/Meath/7/14**  **....................................................................................................** 100

**A/eq/Kilkenny/1/14**  **....................................................................................................** 100

**A/eq/Kilkenny/2/14**  **....................................................................................................** 100

**A/eq/Kilkenny/3/14**  **....................................................................................................** 100

**A/eq/Kilkenny/4/14**  **.....N..............................................................................................** 100

**A/eq/Kilkenny/5/14**  **.....N..............................................................................................** 100

**A/eq/Kilkenny/6/14**  **.....N..............................................................................................** 100

**A/eq/Kilkenny/1/15**  **....................................................................................................** 100

**HA1**

110 120 130 140 150 160 170 180 190 200

....|....|....|....|....|....|....|....|....|....|....|....|....|....|....|....|....|....|....|....|

**A/eq/Meath/1/07**  **DIPDYASLRSIVASSGTLEFTAEGFTWTGVTQNGRSGACKRGSADSFFSRLNWLTKSGNSYPTLNVTMPNNKNFDKLYIWGIHHPSSNQEQTKLYIQESG** 200

**A/eq/Wexford/14**  **..L........I...............................V........................................................** 200

**A/eq/Louth/14**  **..L........I...............................V........................................................** 200

**A/eq/Meath/1/14**  **..L........I...............................V........................................................** 200

**A/eq/Meath/2/14**  **..L........I...............................V........................................................** 200

**A/eq/Meath/3/14**  **..L........I...............................V........................................................** 200

**A/eq/Tipperary/1/14** **..L........I...............................V........................................................** 200

**A/eq/Tipperary/2/14** **..L........I...............................V........................................................** 200

**A/eq/Tipperary/3/14** **..L........I...............................V........................................................** 200

**A/eq/Tipperary/4/14** **..L........I...............................V........................................................** 200

**A/eq/Tipperary/5/14** **..L........I...............................V........................................................** 200

**A/eq/Tipperary/6/14** **..L........I...............................V........................................................** 200

**A/eq/Clare/1/14**  **..L........I...............................V........................................................** 200

**A/eq/Clare/2/14**  **..L........I...............................V........................................................** 200

**A/eq/Clare/3/14**  **..L........I...............................V........................................................** 200

**A/eq/Clare/4/14**  **..L........I...............................V........................................................** 200

**A/eq/Kildare/1/14**  **..L........I...............................V........................................................** 200

**A/eq/Meath/4/14**  **..L........I...............................V........................................................** 200

**A/eq/Meath/5/14**  **..L........I...............................V........................................................** 200

**A/eq/Meath/6/14**  **..L........I...............................V........................................................** 200

**A/eq/Meath/7/14**  **..L........I...............................V........................................................** 200

**A/eq/Kilkenny/1/14**  **..L........I...............................V........................................................** 200

**A/eq/Kilkenny/2/14**  **..L........I...............................V........................................................** 200

**A/eq/Kilkenny/3/14**  **..L........I...............................V........................................................** 200

**A/eq/Kilkenny/4/14**  **..L........I...............................V........................................................** 200

**A/eq/Kilkenny/5/14**  **..L........I...............................V........................................................** 200

**A/eq/Kilkenny/6/14**  **..L........I...............................V........................................................** 200

**A/eq/Kilkenny/1/15**  **..L........I...............................V........................................................** 200

**HA1**

210 220 230 240 250 260 270 280 290 300

....|....|....|....|....|....|....|....|....|....|....|....|....|....|....|....|....|....|....|....|

**A/eq/Meath/1/07**  **RVTVSTKRSQQTIIPNIGSRPWVRGQSGRISIYWTIVKPGDILMINSNGNLVAPRGYFKLKTGKSSVMRSDVPIDICVSECITPNGSISNEKPFQNVNKV** 300

**A/eq/Wexford/14**  **..........................................................................................D........I** 300

**A/eq/Louth/14**  **..........................................................................................D........I** 300

**A/eq/Meath/1/14**  **..........................................................................................D........I** 300

**A/eq/Meath/2/14**  **..........................................................................................D........I** 300

**A/eq/Meath/3/14**  **..........................................................................................D........I** 300

**A/eq/Tipperary/1/14** **..........................................................................................D........I** 300

**A/eq/Tipperary/2/14** **..........................................................................................D........I** 300

**A/eq/Tipperary/3/14** **..........................................................................................D........I** 300

**A/eq/Tipperary/4/14** **..........................................................................................D........I** 300

**A/eq/Tipperary/5/14** **..........................................................................................D........I** 300

**A/eq/Tipperary/6/14** **..........................................................................................D........I** 300

**A/eq/Clare/1/14**  **..........................................................................................D........I** 300

**A/eq/Clare/2/14**  **..........................................................................................D........I** 300

**A/eq/Clare/3/14**  **..........................................................................................D........I** 300

**A/eq/Clare/4/14**  **..........................................................................................D........I** 300

**A/eq/Kildare/1/14**  **..........................................................................................D........I** 300

**A/eq/Meath/4/14**  **..........................................................................................D........I** 300

**A/eq/Meath/5/14**  **.......G..................................................................................D........I** 300

**A/eq/Meath/6/14**  **..........................................................................................D........I** 300

**A/eq/Meath/7/14**  **..........................................................................................D........I** 300

**A/eq/Kilkenny/1/14**  **..........................................................................................D........I** 300

**A/eq/Kilkenny/2/14**  **..........................................................................................D........I** 300

**A/eq/Kilkenny/3/14**  **..........................................................................................D........I** 300

**A/eq/Kilkenny/4/14**  **..........................................................................................D........I** 300

**A/eq/Kilkenny/5/14**  **..........................................................................................D........I** 300

**A/eq/Kilkenny/6/14**  **..........................................................................................D........I** 300

**A/eq/Kilkenny/1/15**  **..........................................................................................D........I** 300

**HA1**

310 320

....|....|....|....|....|....

**A/eq/Meath/1/07**  **TYGKCPKYIRQNTLKLATGMRNVPEKQIR** 329

**A/eq/Wexford/14**  **.............................** 329

**A/eq/Louth/14**  **.............................** 329

**A/eq/Meath/1/14**  **.............................** 329

**A/eq/Meath/2/14**  **.............................** 329

**A/eq/Meath/3/14**  **.............................** 329

**A/eq/Tipperary/1/14** **.............................** 329

**A/eq/Tipperary/2/14** **.............................** 329

**A/eq/Tipperary/3/14** **.............................** 329

**A/eq/Tipperary/4/14** **.............................** 329

**A/eq/Tipperary/5/14** **.............................** 329

**A/eq/Tipperary/6/14** **.............................** 329

**A/eq/Clare/1/14**  **.............................** 329

**A/eq/Clare/2/14**  **.............................** 329

**A/eq/Clare/3/14**  **.............................** 329

**A/eq/Clare/4/14**  **.............................** 329

**A/eq/Kildare/1/14**  **.............................** 329

**A/eq/Meath/4/14**  **.............................** 329

**A/eq/Meath/5/14**  **.............................** 329

**A/eq/Meath/6/14**  **.............................** 329

**A/eq/Meath/7/14**  **.............................** 329

**A/eq/Kilkenny/1/14**  **.............................** 329

**A/eq/Kilkenny/2/14**  **.............................** 329

**A/eq/Kilkenny/3/14**  **.............................** 329

**A/eq/Kilkenny/4/14**  **.............................** 329

**A/eq/Kilkenny/5/14**  **.............................** 329

**A/eq/Kilkenny/6/14**  **.............................** 329

**A/eq/Kilkenny/1/15**  **.............................** 329

**HA2**

10 20 30 40 50 60 70 80 90 100

....|....|....|....|....|....|....|....|....|....|....|....|....|....|....|....|....|....|....|....|

**A/eq/Meath/1/07**  **GIFGAIAGFIENGWEGMVDGWYGFRYQNSEGTGQAADLKSTQTAIDQINEKLNRVIERTNEKFHQIEKEFSEVEGRIQDLEKYVEDTKIDLWSYNAELLV** 100

**A/eq/Wexford/14**  **....................................................................................................** 100

**A/eq/Louth/14**  **....................................................................................................** 100

**A/eq/Meath/1/14**  **....................................................................................................** 100

**A/eq/Meath/2/14**  **....................................................................................................** 100

**A/eq/Meath/3/14**  **....................................................................................................** 100

**A/eq/Tipperary/1/14** **....................................................................................................** 100

**A/eq/Tipperary/2/14** **....................................................................................................** 100

**A/eq/Tipperary/3/14** **....................................................................................................** 100

**A/eq/Tipperary/4/14** **....................................................................................................** 100

**A/eq/Tipperary/5/14** **....................................................................................................** 100

**A/eq/Tipperary/6/14** **....................................................................................................** 100

**A/eq/Clare/1/14**  **....................................................................................................** 100

**A/eq/Clare/2/14**  **....................................................................................................** 100

**A/eq/Clare/3/14**  **....................................................................................................** 100

**A/eq/Clare/4/14**  **....................................................................................................** 100

**A/eq/Kildare/1/14**  **....................................................................................................** 100

**A/eq/Meath/4/14**  **....................................................................................................** 100

**A/eq/Meath/5/14**  **....................................................................................................** 100

**A/eq/Meath/6/14**  **....................................................................................................** 100

**A/eq/Meath/7/14**  **....................................................................................................** 100

**A/eq/Kilkenny/1/14**  **....................................................................................................** 100

**A/eq/Kilkenny/2/14**  **....................................................................................................** 100

**A/eq/Kilkenny/3/14**  **....................................................................................................** 100

**A/eq/Kilkenny/4/14**  **....................................................................................................** 100

**A/eq/Kilkenny/5/14**  **....................................................................................................** 100

**A/eq/Kilkenny/6/14**  **....................................................................................................** 100

**A/eq/Kilkenny/1/15**  **....................................................................................................** 100

**HA2**

110 120 130 140 150 160 170 180 190 200

....|....|....|....|....|....|....|....|....|....|....|....|....|....|....|....|....|....|....|....|

**A/eq/Meath/1/07**  **ALENQHTIDLTDAEMNKLFEKTRRQLRENAEDMGGGCFKIYHKCDNACIGSIRNGTYDHYIYRDEALNNRFQIKGVELKSGYKDWILWISFAISCFLICV** 200

**A/eq/Wexford/14**  **............T.........................................................................M.............** 200

**A/eq/Louth/14**  **............T.........................................................................M.............** 200

**A/eq/Meath/1/14**  **......................................................................................M.............** 200

**A/eq/Meath/2/14**  **......................................................................................M.............** 200

**A/eq/Meath/3/14**  **......................................................................................M.............** 200

**A/eq/Tipperary/1/14** **......................................................................................M...........Y.** 200

**A/eq/Tipperary/2/14** **......................................................................................M...........Y.** 200

**A/eq/Tipperary/3/14** **......................................................................................M.............** 200

**A/eq/Tipperary/4/14** **......................................................................................M.............** 200

**A/eq/Tipperary/5/14** **......................................................................................M.............** 200

**A/eq/Tipperary/6/14** **......................................................................................M.............** 200

**A/eq/Clare/1/14**  **......................................................................................M.............** 200

**A/eq/Clare/2/14**  **......................................................................................M.............** 200

**A/eq/Clare/3/14**  **......................................................................................M.............** 200

**A/eq/Clare/4/14**  **......................................................................................M.............** 200

**A/eq/Kildare/1/14**  **......................................................................................M.............** 200

**A/eq/Meath/4/14**  **......................................................................................M.............** 200

**A/eq/Meath/5/14**  **......................................................................................M.............** 200

**A/eq/Meath/6/14**  **......................................................................................M.............** 200

**A/eq/Meath/7/14**  **......................................................................................M.............** 200

**A/eq/Kilkenny/1/14**  **......................................................................................M.............** 200

**A/eq/Kilkenny/2/14**  **......................................................................................M.............** 200

**A/eq/Kilkenny/3/14**  **......................................................................................M.............** 200

**A/eq/Kilkenny/4/14**  **......................................................................................M.............** 200

**A/eq/Kilkenny/5/14**  **......................................................................................M.............** 200

**A/eq/Kilkenny/6/14**  **......................................................................................M.............** 200

**A/eq/Kilkenny/1/15**  **......................................................................................M.............** 200

**HA2**

210 220

....|....|....|....|.

**A/eq/Meath/1/07**  **VLLGFIMWACQKGNIRCNICI** 221

**A/eq/Wexford/14**  **.....................** 221

**A/eq/Louth/14**  **.....................** 221

**A/eq/Meath/1/14**  **...S.................** 221

**A/eq/Meath/2/14**  **...S.................** 221

**A/eq/Meath/3/14**  **...S.................** 221

**A/eq/Tipperary/1/14** **.....................** 221

**A/eq/Tipperary/2/14** **.....................** 221

**A/eq/Tipperary/3/14** **.....................** 221

**A/eq/Tipperary/4/14** **.....................** 221

**A/eq/Tipperary/5/14** **.....................** 221

**A/eq/Tipperary/6/14** **.....................** 221

**A/eq/Clare/1/14**  **.....................** 221

**A/eq/Clare/2/14**  **.....................** 221

**A/eq/Clare/3/14**  **.....................** 221

**A/eq/Clare/4/14**  **.....................** 221

**A/eq/Kildare/1/14**  **.....................** 221

**A/eq/Meath/4/14**  **.....................** 221

**A/eq/Meath/5/14**  **.....................** 221

**A/eq/Meath/6/14**  **.....................** 221

**A/eq/Meath/7/14**  **.....................** 221

**A/eq/Kilkenny/1/14**  **.....................** 221

**A/eq/Kilkenny/2/14**  **.....................** 221

**A/eq/Kilkenny/3/14**  **.....................** 221

**A/eq/Kilkenny/4/14**  **.....................** 221

**A/eq/Kilkenny/5/14**  **.....................** 221

**A/eq/Kilkenny/6/14**  **.....................** 221

**A/eq/Kilkenny/1/15**  **.....................** 221
