# Supplementary material for: Multifocal Equine Influenza Outbreak with Vaccination Breakdown in Thoroughbred Racehorses
Source: Pathogens. 2018 Apr 17;7(2):43. doi: 10.3390/pathogens7020043 (PMC6027538; doi:10.3390/pathogens7020043)
Supplement: Supplementary file 1 [file pathogens-07-00043-s001.zip › SupplementaryData/Supplementary Figure S2.docx]

**Supplementary Figure S2: NA amino acid alignment.** Alignment of the predicted NA amino acid sequences of Irish FCL2 strains (2008-2014) and FCL1 virus (A/eq/Limerick/3/10) against reference strain A/eq/Meath/1/07. Amino acid identity is represented with a dot.

10 20 30 40 50 60 70 80 90 100

....|....|....|....|....|....|....|....|....|....|....|....|....|....|....|....|....|....|....|....|

**A/eq/Meath/1/07**  **MNPNQKIITIGSASLGILIINVILHVVSIIVTVLVLNNNETGLNCKGTIIREYNETVRVEKITQWHNTSAIKYIERPPNEYYMNNTEPLCEAQGFAPFSK** 100

**A/eq/Down/1/08**  **....................................................................................................** 100

**A/eq/Limerick/3/10**  **.....................I..............................................................................** 100

**A/eq/Kildare/4/10**  **....................................................................................................** 100

**A/eq/Carlow/11**  **....................................................................................................** 100

**A/eq/Kildare/2/12**  **........................N...........................................................................** 100

**A/eq/Kilkenny/1/12**  **....................................................................................................** 100

**A/eq/Meath/1/14**  **........................N................C..........................................................** 100

**A/eq/Tipperary/1/14** **........................N................C..........................................................** 100

**A/eq/Tipperary/3/14** **........................N................C..........................................................** 100

**A/eq/Kildare/1/14**  **.....................I..N................C..........................................................** 100

**A/eq/Clare/2/14**  **........................N................C..........................................................** 100

**A/eq/Kilkenny/1/14**  **...........F............N................C..........................................................** 100

**A/eq/Kilkenny/4/14**  **...........F............N................C..........................................................** 100

**A/eq/Meath/4/14**  **........................N................C..........................................................** 100

**A/eq/Meath/5/14**  **........................N................C..........................................................** 100

110 120 130 140 150 160 170 180 190 200

....|....|....|....|....|....|....|....|....|....|....|....|....|....|....|....|....|....|....|....|

**A/eq/Meath/1/07**  **DNGIRIGSRGHVFVIREPFVSCSPSECRTFFLTQGSLLNDKHSNGTVKDRSPYRTLMSVKIGQSPNVYQARFESVAWSATACHDGKKWMTIGVTGPDNQA** 200

**A/eq/Down/1/08**  **........K...........................................................................................** 200

**A/eq/Limerick/3/10**  **....................................................................................................** 200

**A/eq/Kildare/4/10**  **........K...........................................................................................** 200

**A/eq/Carlow/11**  **........K...........................................................................................** 200

**A/eq/Kildare/2/12**  **........K...........................................................................................** 200

**A/eq/Kilkenny/1/12**  **........K...........................................................................................** 200

**A/eq/Meath/1/14**  **........K...........................................................................................** 200

**A/eq/Tipperary/1/14** **........K...........................................................................................** 200

**A/eq/Tipperary/3/14** **........K...........................................................................................** 200

**A/eq/Kildare/1/14**  **........K...........................................................................................** 200

**A/eq/Clare/2/14**  **........K...........................................................................................** 200

**A/eq/Kilkenny/1/14**  **........K...........................................................................................** 200

**A/eq/Kilkenny/4/14**  **........K...........................................................................................** 200

**A/eq/Meath/4/14**  **........K...........................................................................................** 200

**A/eq/Meath/5/14**  **........K...........................................................................................** 200

**NA**

210 220 230 240 250 260 270 280 290 300

....|....|....|....|....|....|....|....|....|....|....|....|....|....|....|....|....|....|....|....|

**A/eq/Meath/1/07**  **IAVVNYGGVPVDIINSWAGDILRTQESSCTCIKGNCYWVMTDGPANRQAKYRIFKAKDGRVIGQTDISFNGGHIEECSCYPNEGKVECICRDNWTGTNRP** 300

**A/eq/Down/1/08**  **....................................................................................................** 300

**A/eq/Limerick/3/10**  **....................................................................................................** 300

**A/eq/Kildare/4/10**  **....................................................................................................** 300

**A/eq/Carlow/11**  **....................................................................................................** 300

**A/eq/Kildare/2/12**  **....................................................................................................** 300

**A/eq/Kilkenny/1/12**  **....................................................................................................** 300

**A/eq/Meath/1/14**  **....................................................................................................** 300

**A/eq/Tipperary/1/14** **....................................................................................................** 300

**A/eq/Tipperary/3/14** **....................................................................................................** 300

**A/eq/Kildare/1/14**  **....................................................................................................** 300

**A/eq/Clare/2/14**  **....................................................................................................** 300

**A/eq/Kilkenny/1/14**  **....................................................................................................** 300

**A/eq/Kilkenny/4/14**  **....................................................................................................** 300

**A/eq/Meath/4/14**  **....................................................................................................** 300

**A/eq/Meath/5/14**  **....................................................................................................** 300

310 320 330 340 350 360 370 380 390 400

....|....|....|....|....|....|....|....|....|....|....|....|....|....|....|....|....|....|....|....|

**A/eq/Meath/1/07**  **ILVISSDLSYTVGYLCAGIPTDTPRGEDSQFTGSCTSPLGNKGYGVKGFGFRQGTDVWAGRTISRTSRSGFEIIKIRNGWTQNSKDQIRRQVIIDDPNWS** 400

**A/eq/Down/1/08**  **....................................................................................................** 400

**A/eq/Limerick/3/10**  **....................................................................................................** 400

**A/eq/Kildare/4/10**  **....................................................................................................** 400

**A/eq/Carlow/11**  **....................................................................................................** 400

**A/eq/Kildare/2/12**  **....................................................................................................** 400

**A/eq/Kilkenny/1/12**  **....................................................................................................** 400

**A/eq/Meath/1/14**  **....................................................................................................** 400

**A/eq/Tipperary/1/14** **....................................................................................................** 400

**A/eq/Tipperary/3/14** **....................................................................................................** 400

**A/eq/Kildare/1/14**  **....................................................................................................** 400

**A/eq/Clare/2/14**  **....................................................................................................** 400

**A/eq/Kilkenny/1/14**  **....................................................................................................** 400

**A/eq/Kilkenny/4/14**  **....................................................................................................** 400

**A/eq/Meath/4/14**  **....................................................................................................** 400

**A/eq/Meath/5/14**  **....................................................................................................** 400

**NA**

410 420 430 440 450 460 470

....|....|....|....|....|....|....|....|....|....|....|....|....|....|

**A/eq/Meath/1/07**  **GYSGSFTLPIELTKKGCLVPCFWVEMIRGKPEETTIWTSSSSIVMCGVDHKIASWSWHDGAILPFDIDKM** 470

**A/eq/Down/1/08**  **.........V............................................................** 470

**A/eq/Limerick/3/10**  **.........V........................................E...................** 470

**A/eq/Kildare/4/10**  **.........V....R..................S....................................** 470

**A/eq/Carlow/11**  **.........V....R..................S....................................** 470

**A/eq/Kildare/2/12**  **.........V....R..................S....................................** 470

**A/eq/Kilkenny/1/12**  **.........V....R..................S....................................** 470

**A/eq/Meath/1/14**  **.........V....R..................S....................................** 470

**A/eq/Tipperary/1/14** **.........V....R..................S....................................** 470

**A/eq/Tipperary/3/14** **.........V....R..................S....................................** 470

**A/eq/Kildare/1/14**  **.........V....R..................S....................................** 470

**A/eq/Clare/2/14**  **.........V....R..................S....................................** 470

**A/eq/Kilkenny/1/14**  **.........V....R..................S....................................** 470

**A/eq/Kilkenny/4/14**  **.........V....R..................S....................................** 470

**A/eq/Meath/4/14**  **.........V....R..................S....................................** 470

**A/eq/Meath/5/14**  **.........V....R..................S....................................** 470
