# Supplementary material for: Multifocal Equine Influenza Outbreak with Vaccination Breakdown in Thoroughbred Racehorses
Source: Pathogens. 2018 Apr 17;7(2):43. doi: 10.3390/pathogens7020043 (PMC6027538; doi:10.3390/pathogens7020043)
Supplement: Supplementary file 1 [file pathogens-07-00043-s001.zip › SupplementaryData/Supplementary Table S1.docx]

**Supplementary Table S1: Equine influenza viruses included in phylogenetic analysis**

| **Location** | **Virus Name** | **HA1 accession number** | **NA accession number** |
| --- | --- | --- | --- |
| Miami, USA | A/eq/Miami/63 | M29257 | N/A |
| Miami, USA | A/eq/Miami/1/63 | N/A | CY028838 |
| Fontainebleu, France | A/eq/Fontainebleu/1/79 | CY032405 | CY032407 |
| California, USA | A/eq/California/103/82 | N/A | CY028910 |
| Kildare, Ireland | A/eq/Kildare/1/89 | JN222941 | N/A |
| Sussex, UK | A/eq/Sussex/1/89 | N/A | CY032319 |
| Berlin, Germany | A/eq/Berlin/1/89 | N/A | CY032415 |
| Hong Kong | A/eq/Hong Kong/1/92 | L27597 | N/A |
| Austria | A/eq/Austria/421/92 | N/A | CY032351 |
| Kildare, Ireland | A/eq/Kildare/1/92 | JN084402 | N/A |
| Newmarket, UK | A/eq/Newmarket/1/93 | X85088 | FJ375222 |
| Newmarket, UK | A/eq/Newmarket/2/93 | X85089 | FJ375223 |
| Kentucky, USA | A/eq/Kentucky/8/94 | N/A | CY030183 |
| Kentucky, USA | A/eq/Kentucky/1/97 | AF197249 | N/A |
| Kentucky, USA | A/eq/Kentucky/1/98 | AF197241 | N/A |
| Ohio, USA | A/eq/Ohio/1/03 | DQ124192 | DQ124168 |
| Newmarket, UK | A/eq/Newmarket/5/03 | FJ375213 | N/A |
| South Africa | A/eq/South Africa/4/03 | GU447312 | N/A |
| Kentucky, USA | A/eq/Kentucky/9/04 | FJ195451 | N/A |
| Aboyne, Scotland | A/eq/Aboyne/1/05 | EF541442 | N/A |
| Aboyne, Scotland | A/eq/Aboyne/05 | N/A | KF049177 |
| Switzerland | A/eq/Switzerland/P112/07 | FJ195408 | N/A |
| Sydney, Australia | A/eq/Sydney/6085/07 | GU045763 | N/A |
| Meath, Ireland | A/eq/Meath/1/07 | JN222935 | MG586816 |
| Richmond, UK | A/eq/Richmond/1/07 | FJ195395 | KF559336 |
| Ibaraki, Japan | A/eq/Ibaraki/1/07 | AB360549 | AB360608 |
| Lincolnshire, UK | A/eq/Lincolnshire/1/07 | FJ195398 | KF559342 |
| Down, Ireland | A/eq/Down/1/08 | JN222937 | MG586817 |
| Donegal, Ireland | A/eq/Donegal/1/09 | JN222938 | N/A |
| Carlow, Ireland | A/eq/Carlow/1/09 | JN222939 | N/A |
| Limerick, Ireland | A/eq/Limerick/1/10 | JN222940 | N/A |
| Limerick, Ireland | A/eq/Limerick/3/10 | N/A | MG586818 |
| Kildare, Ireland | A/eq/Kildare/2/10 | KC871537 | N/A |
| Kildare, Ireland | A/eq/Kildare/4/10 | N/A | MG586819 |
| Kilkenny, Ireland | A/eq/Kilkenny/11 | KC871545 | N/A |
| Carlow, Ireland | A/eq/Carlow/11 | KC871546 | MG586820 |
| Mongolia | A/eq/Mongolia/6/11 | JX549062 | N/A |
| New York, USA | A/eq/New York/1/11 | KF026399 | N/A |
| East Renfrewshire, UK | A/eq/East Renfrewshire/1/11 | KF049198 | N/A |
| East Renfrewshire, UK | A/eq/East Renfrewshire/2/11 | N/A | KF049172 |
| Devon, UK | A/eq/Devon/1/11 | N/A | KF049194 |
| Kilkenny, Ireland | A/eq/Kilkenny/1/12 | N/A | MG586822 |
| Kilkenny, Ireland | A/eq/Kilkenny/3/12 | KC871549 | N/A |
| Kildare, Ireland | A/eq/Kildare/2/12 | KC871557 | MG586821 |
| Lichtenfeld, Germany | A/eq/Lichtenfeld/1/12 | JX499136 | KF049191 |
| County Durham, UK | A/eq/County Durham/2/12 | KF026396 | KF049192 |
| Rastatt, Germany | A/eq/Rastatt/1/12 | KC584975 | N/A |
| Dubai, UAE | A/eq/Dubai/3/12 | KF026413 | N/A |
| Dubai, UAE | A/eq/Dubai/1/12 | N/A | KF049173 |
| Argentina | A/eq/Argentina/E-2345-1/12 | KJ372713 | N/A |
| South Kazakhstan | A/eq/South Kazakhstan/236/12 | KF712451 | N/A |
| Kentucky, USA | A/eq/Kentucky/4/12 | N/A | KF049185 |
| Heilongjiang, China | A/eq/Heilongjiang/SS1/13 | KC986390 | KC986392 |
| North Rhine Westphalia, Germany | A/eq/North Rhine Westphalia/1/14 | KJ538149 | N/A |
| Wexford, Ireland | A/eq/Wexford/14 | MG586802 | N/A |
| Louth, Ireland | A/eq/Louth/14 | MG586803 | N/A |
| Meath, Ireland | A/eq/Meath/1/14 | MG586804 | MG586823 |
| Tipperary, Ireland | A/eq/Tipperary/1/14 | MG586805 | MG586824 |
| Tipperary, Ireland | A/eq/Tipperary/3/14 | MG586806 | MG586825 |
| Clare, Ireland | A/eq/Clare/1/14 | MG586807 | N/A |
| Kildare, Ireland | A/eq/Kildare/1/14 | MG586808 | MG586826 |
| Clare, Ireland | A/eq/Clare/2/14 | MG586809 | MG586827 |
| Kilkenny, Ireland | A/eq/Kilkenny/1/14 | MG586810 | MG586828 |
| Kilkenny, Ireland | A/eq/Kilkenny/4/14 | MG586811 | MG586829 |
| Meath, Ireland | A/eq/Meath/4/14 | MG586812 | MG586830 |
| Meath, Ireland | A/eq/Meath/5/14 | MG586813 | MG586831 |
| Meath, Ireland | A/eq/Meath/6/14 | MG586814 | N/A |
| Kilkenny, Ireland | A/eq/Kilkenny/1/15 | MG586815 | N/A |
